# Assessing Circulating Tumour DNA (ctDNA) as a Biomarker for Anal Cancer Management: A Systematic Review

**DOI:** 10.3390/ijms25074005

**Published:** 2024-04-03

**Authors:** Hugo C. Temperley, Timothy Fannon, Niall J. O’Sullivan, Maeve O’Neill, Benjamin M. Mac Curtain, Charles Gilham, Jacintha O’Sullivan, Grainne O’Kane, Brian J. Mehigan, Sharon O’Toole, John O. Larkin, David Gallagher, Paul McCormick, Michael E. Kelly

**Affiliations:** 1 Department of Radiology, St. James’s Hospital, D08 NHY1 Dublin, Ireland; temperlh@tcd.ie (H.C.T.);; 2 Department of Surgery, St. James’s Hospital, D08 NHY1 Dublin, Ireland; 3 Trinity St. James’s Cancer Institute, D08 NHY1 Dublin, Ireland; 4 Department of Radiation Oncology, St. James’s Hospital, D08 NHY1 Dublin, Ireland; 5 Trinity Translational Medicine Institute, Trinity St. James’s Cancer Institute, Trinity College, St. James’s Hospital, D08 NHY1 Dublin, Ireland; 6 Department of Medical Oncology, St. James’s Hospital, D08 NHY1 Dublin, Ireland; 7 Department of Genetics, St. James’s Hospital, D08 NHY1 Dublin, Ireland

**Keywords:** ctDNA, anal cancer, salvage surgery, translational oncology, surgical oncology

## Abstract

This systematic review investigates the potential of circulating tumour DNA (ctDNA) as a predictive biomarker in the management and prognosis of squamous cell carcinoma of the anal canal (SCCA). PubMed, EMBASE, and Cochrane Central Registry of Controlled Trials were searched until 7 January 2024. Selection criteria included research articles exploring ctDNA in the context of anal cancer treatment response, recurrence risk assessment, and consideration of salvage surgery. A total of eight studies were therefore included in the final review, examining a total of 628 patients. These studies focused on three main themes: SCCA diagnosis and staging, treatment response, and patient outcomes. Significant heterogeneity was observed in terms of patient cohort, study methodology, and ctDNA biomarkers. Four studies provided information on the sensitivity of ctDNA biomarkers in SCCA, with a range of 82–100%. Seven studies noted a correlation between pre-treatment ctDNA levels and SCCA disease burden, suggesting that ctDNA could play a role as a biomarker for the staging of SCCA. Across all seven studies with paired pre- and post-treatment ctDNA samples, a trend was seen towards decreasing ctDNA levels post-treatment, with specific identification of a ‘fast elimination’ group who achieve undetectable ctDNA levels prior to the end of treatment and may be less likely to experience treatment failure. Residual ctDNA detection post-treatment was associated with poorer patient prognosis. This systematic review identifies the broad potential of ctDNA as a useful and decisive tool in the management of SCCA. Further analysis of ctDNA biomarkers that include larger patient cohorts is required in order to clearly evaluate their potential role in clinical decision-making processes.

## 1. Introduction

In recent years, there has been a paradigm shift in cancer research towards precision medicine, aiming to tailor therapeutic approaches based on the molecular characteristics of individual tumours [1]. One promising avenue in this endeavour is the exploration of circulating tumour DNA (ctDNA) as a biomarker in cancer management [2]. Squamous cell carcinoma of the anal canal (SCCA), a relatively rare malignancy with distinct challenges in treatment and surveillance, has become a focus of investigation for the potential utility of ctDNA [3,4,5].

ctDNA, also known as liquid biopsy, refers to small fragments of DNA in the bloodstream that originate from tumour cells [6]. This biological material carries genetic information reflective of the tumour’s genome. The unique advantage of ctDNA lies in its accessibility through simple phlebotomy, circumventing the need for invasive procedures [6]. These fragments, originating from both primary and metastatic lesions, offer a real-time snapshot of the tumour’s genetic heterogeneity [7]. As the field of oncology progresses towards personalised treatment strategies, ctDNA has emerged as a promising tool for capturing the intricacies of tumour biology, enabling clinicians to make informed decisions about therapeutic interventions [8]. 

The incorporation of ctDNA analysis into the landscape of SCCA management holds significant potential. Chemoradiation therapy (CRT) is the primary modality for treating localised SCCA, but predicting treatment response can be difficult [9]. ctDNA may serve as a dynamic biomarker, reflecting the evolving genomic landscape during and after treatment. Identification of specific genetic alterations in ctDNA might not only predict the efficacy of CRT but also guide the selection of targeted therapies for personalised treatment strategies.

Furthermore, the role of ctDNA in surveillance, highlighting those at risk of requiring salvage surgery, is attractive. Salvage surgery, in the form of abdominoperineal resection, becomes a crucial consideration in cases of persistent or recurrent SCCA after initial CRT in order to achieve local control and improve survival. It is important to consider the morbidities of major surgery when considering this treatment modality [10]. The detection of elevated ctDNA levels or specific genetic alterations may serve as an indicator of treatment failure, prompting timely decisions regarding salvage surgery. Understanding the correlation between ctDNA dynamics and the likelihood of salvage surgery requirement is essential for optimising patient outcomes and minimising unnecessary interventions. This systematic review will explore the existing literature to provide insights into the evolving landscape of ctDNA-guided decisions in salvage surgery for SCCA, offering a comprehensive overview of the current state of knowledge in this domain.

## 2. Methods

### 2.1. Registration and Search Strategy

**Figure 1:**
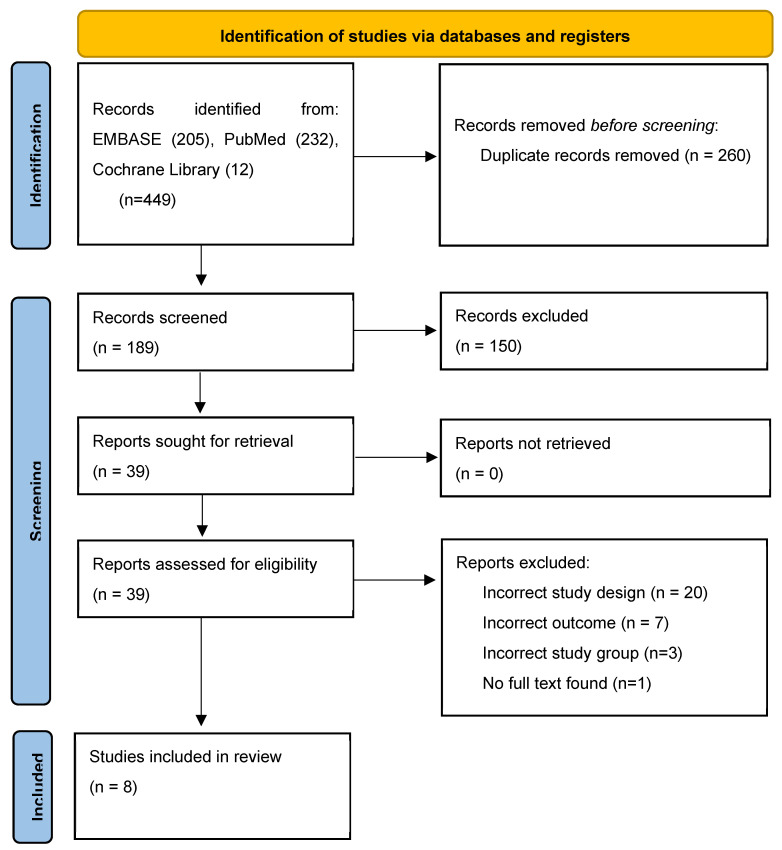
PRISMA statement for ctDNA in anal cancer.

Our search was conducted in line with the most recent Preferred Reporting Items for Systematic Reviews and Meta-Analyses (PRISMA) recommendations [11]. Our study protocol was prospectively registered with PROSPERO (515992). We conducted a search using PubMed, EMBASE, and Cochrane Central Register of Controlled Trials up to 7 January 2024. The search pathway has been illustrated in the PRISMA diagram in Figure 1. The grey literature (information available outside of traditional academic publishing) was also searched for any relevant studies. The systematic search process with detailed search terms is outlined in Supplementary Material S1. Due to heterogeneity and the descriptive nature in which the results were presented, a narrative summary of findings is presented, and meta-analysis was not appropriate.

### 2.2. Inclusion/Exclusion Criteria

#### 2.2.1. Inclusion Criteria

In order to be included in our analysis, the studies had to meet the following criteria:

(a) Study evaluated the role of ctDNA in the context of SCCA management.
(b) Study included patients aged 18 years or older.
(c) Full-text manuscript was available.

#### 2.2.2. Exclusion Criteria

Studies were excluded from the analysis if:

(a) Patients did not have a diagnosis of SCCA.
(b) Outcomes of interest were not reported.
(c) The study methodology was not clearly reported.

### 2.3. Outcomes of Interest

Studies that satisfied the inclusion and exclusion criteria were included in our review. Information extracted was based on the PICO framework (Population, Intervention, Comparator, and Outcomes) [11]. The following PICO elements were used as the basis for selecting studies:

I. Participants: studies that involved a human subject of any age who was diagnosed with an anal carcinoma as a primary lesion or as a relapse.
II. Intervention: detection of ctDNA and CTCs (circulating tumour cells) and/or cHPV (human papillomavirus) DNA in plasma or serum of participants before, during, and after treatment.
III. Comparisons: healthy controls, cytology/histology results of tissue samples from tumours or any tumour biomarker results before and/or after treatment.
IV. Outcome measure: the accuracy of ctDNA and/or cHPV DNA in the diagnosis, monitoring, and prediction of relapse of SCCA. This included the sensitivity, specificity, positive predictive value, and negative predictive value of ctDNA/cHPV DNA and the diagnostic odds ratio as a diagnostic/prognostic tool for the detection of SCCA. The gold standard for diagnosis was (as a minimum) histological confirmation of the cancer.
  (a) predicting responses to CRT
  (b) assessing recurrence risk
  (c) guiding salvage surgery decisions
V. Additional outcome(s): qualitative analysis summarising detection techniques, results, and clinical observations in cases where accuracy (sensitivity/specificity) data were not available.
VI. Types of studies: the review considered all studies evaluating the effectiveness or efficacy of interventions.

### 2.4. Study Selection, Data Extraction, and Critical Appraisal

A database was created using the reference managing software EndNote X9 TM Version 2.0 (Clarivate, London). Abstracts of articles yielded from the search were reviewed by two independent authors (HCT and NOS) based on the inclusion and exclusion criteria detailed above. Following the removal of duplicate articles, discrepancies in judgment about the relevance of articles were resolved via an open discussion between the authors and an independent third reviewer (MK). An article was excluded from the review when the three reviewers came to an agreement. The full texts of short-listed articles were obtained and further evaluated to ensure that they met our inclusion criteria. The references of short-listed articles were then searched to identify other relevant studies that may have been missed through the initial search of online databases. Data were extracted by two reviewers independently from the articles that met inclusion criteria based on full-text review. In order to extract and store data efficiently, the Cochrane Collaboration screening and data extraction tool Covidence (Veritas Health Innovation, Melbourne, Australia. Available at www.covidence.org (accessed on 20 January 2024)) was used.

### 2.5. Risk of Bias

Potential biases were assessed using the Newcastle-Ottawa scale (HT) risk of bias tool and the results were tabulated [12]. This assessment tool grades each study as being ‘satisfactory’ or ‘unsatisfactory’ across various categories. We assigned stars to evaluate study quality: 7 stars—“very good”, 5–6 stars “good”, 3–4 stars “satisfactory”, and 0–2 stars “unsatisfactory”. The critical appraisal was completed by two reviewers independently (HT & NOS), where, once again, a third reviewer (MK) was asked to arbitrate in cases of discrepancies in opinion.

## 3. Results

### 3.1. Search Results

The initial search identified 449 studies (Figure 1), of which 260 duplicates were removed. Titles and abstracts were screened for 189, of which 150 studies did not meet eligibility criteria. Of the remaining thirty-nine studies, twenty-four studies did not report on ctDNA/cHPV DNA, three were review articles, one was a case report, and one did not focus on anogenital cancer. A total of eight studies were therefore included in the final review [13,14,15,16,17,18,19,20].

### 3.2. Study Characteristics

The eight included studies were published between 2018 and 2023 [13,14,15,16,17,18,19,20]. The number of patients in each individual study ranged from 15 to 251, with a total of 628 patients across the eight studies. Disease stage inclusion criteria varied between studies, from anal HSIL (high-grade squamous intra-epithelial lesion) (n = 1) to SCCA stages I-IV (n = 3). DNA biomarkers and detection methods also varied. Six studies assessed the use of ctDNA [13,14,15,16,18,20], of which five examined HPV ctDNA, and one examined multiple single nucleotide variants in ctDNA [13]. The remaining two studies assessed HPV cell-free DNA and circulating free DNA, respectively [17,19]. One study was qualitative in their detection of DNA biomarkers [18], while the remaining seven were quantitative. Four studies used droplet digital PCR (ddPCR) for biomarker assessment [14,15,16,19], and 7/8 studies measured DNA biomarkers both pre- and post-treatment [13,14,15,16,17,18,20]. Treatment consisted of either CRT (n = 6) or chemotherapy alone (n = 1). Further information regarding study characteristics is included in Table 1.

### 3.3. ctDNA as a Diagnostic Biomarker in SCCA

Four studies provided information on the sensitivity and/or specificity of ctDNA as a biomarker in SCCA [14,15,16,19]. Lefevre et al. (2021) measured plasma HPV DNA in a cohort of 88 patients with stages I-IV SCCA prior to treatment [17]. In patients with HPV-related tumours, this study observed a plasma HPV DNA sensitivity of 82% and specificity of 67% for SCCA. Cabel et al. found that HPV ctDNA had a sensitivity of 88% in their cohort of 33 patients with stage II–III HPV16- or HPV18-positive SCCA prior to treatment (95% CI: 72–95%) [15]. Bernard-Tessier et al. detected HPV ctDNA in 52/57 patients with stages III–IV HPV16-related SCCA prior to treatment, indicating a sensitivity of 91.1% (95% CI: 81.1–96.2%) [14]. To investigate the potential use of HPV cell-free DNA as a screening tool for SCCA, Ellsworth et al. conducted a prospective study of 40 HIV patients with either HPV infection (n = 20) or anal HSIL (high-grade squamous intraepithelial lesions) (n = 20), with the hypothesis that no HPV cell-free DNA would be detected in either patient cohort [19]. Despite HPV16 being detected in 10 anal swab specimens and 5 HSIL biopsy specimens, no patients in either cohort had droplet detection of HPV16 cell-free DNA from plasma, therefore demonstrating a specificity of 100% for detection of invasive cancer (95% CI: 91–100%).

Six studies observed a correlation between quantitative measurement of ctDNA and SCCA disease burden prior to treatment [13,14,15,16,17,18]. Overall, 73/88 patients included in Lefevre et al. (2021) had plasma HPV DNA levels quantitatively measured prior to treatment [17]. In this study, median pre-treatment plasma HPV DNA levels increased with advancing tumour stage; however, these differences did not reach statistical significance. There was a significantly higher plasma HPV DNA level in patients with lymph node-positive versus lymph node-negative disease: 6.09% (95% CI: 2.08–34.54) and 0.39% (95% CI: 0.18–2.59), respectively (*p* = 0.02). Overall, 73/80 patients included in Lefevre et al. (2020) had circulating free DNA levels quantitatively measured prior to treatment [16]. Similarly, the median circulating free DNA levels increased with advancing tumour stage but did not reach statistical significance. Baseline circulating free DNA levels did, however, correlate with gross tumour volume for all patients (R^2^ adjusted of 0.13, *p* < 0.01). In their cohort of 59 patients with stages III–IV SCCA, Bernard-Tessier et al. saw a correlation between tumour burden and baseline HPV ctDNA levels prior to treatment (Spearman r = 0.32 (95% CI: 0.03–0.6), *p* = 0.025) in addition to significantly higher levels of HPV ctDNA in patients with metastatic disease versus loco-regional recurrence [14]. In a cohort of 62 patients with SCCA, Mazurek et al. used a multiple regression model to identify a correlation between node positive disease and a high circulating tumour-related HPV16 DNA viral load (*p* = 0.031) [18]. Similarly, Cabel et al. observed a correlation between HPV ctDNA levels and lymph node status, with a median ctDNA level of 85 copies/mL (range: 8.7–9.333) in node-positive SCCA versus 32 copies/mL (range: 3–1350) in node-negative SCCA (*p* = 0.03) [15]. Azzi et al. observed that patients with metastatic disease had significantly higher levels of ctDNA than those with localised disease (*p* = 0.004) [13]. Furthermore, Azzi et al. did not observe any significant difference in ctDNA levels between patients with stage I, II, or III disease [13]. See Table 2 for a summary of the role of ctDNA in SCCA diagnosis and staging.

### 3.4. ctDNA and Treatment Response

Seven studies out of a total of eight included studies assessed the role of ctDNA as an indicator of treatment response in SCCA [13,14,15,16,17,18,20]. ctDNA measurement timepoints included pre-treatment, mid-treatment, post-treatment (end of treatment), and extended post-treatment follow-up. Six studies assessed ctDNA in patients with SCCA following CRT [13,15,16,17,18,20]. One of these studies also incorporated patients who received radiotherapy, immunotherapy, or surgery as alternative treatment modalities [13]. One study assessed patients following chemotherapy alone [14]. Data pertaining to follow-up timepoints and number of patients included at each follow-up timepoint in each study have been included in Table 1. Three studies evaluated treatment response using RECIST (Response Evaluation Criteria in Solid Tumours) criteria [13,14,18]. One study evaluated treatment response using combined clinical and radiographic methods [15]. Four studies did not document their method for evaluating treatment response [16,17,19,20].

Paired samples of HPV ctDNA were available following CRT in 18 patients included in Cabel et al., and only three of which had detectable residual HPV ctDNA levels after treatment (detection rate = 17%, 95% CI: 5.8–39) [15]. Neither pre-treatment ctDNA detection nor pre-treatment ctDNA levels were associated with treatment failure (*p* = 0.26, *p* = 0.77, respectively). Three of eighteen patients with paired plasma samples pre- and post-CRT were shown to have residual HPV ctDNA levels following treatment. These patients went on to experience rapid metastatic relapse. One of fifteen patients who were HPV ctDNA-negative post-treatment went on to experience local relapse after 8.5 months (7%, 95% CI: 1–30). Lefevre et al. (2020) recorded the highest median level of circulating free DNA prior to treatment, with a decreasing median circulating free DNA level from baseline to a mid-therapy timepoint (0.71 copies/mL, *p* < 0.01) and from baseline to one-year follow-up (0.71 copies/mL, *p* < 0.01) [16]. No correlation was observed between changes in circulating free DNA during CRT and disease prognosis. All patients who experienced relapse after a median follow-up period of 22 months in this study had baseline circulating free DNA levels over the 25th percentile (*p* = 0.05).

Mazurek et al. measured circulating tumour-related HPV16 DNA pre-treatment, mid-treatment, post-treatment, and at three further timepoints over the following 3 years [18]. In 19/21 patients for whom follow-up data were available, a decrease in ctHPV16 DNA viral load to undetectable levels was observed either mid-treatment (n = 8), post-treatment (n = 6), 1–8 months post-treatment (n = 4), or by 14 months post-treatment (n = 1). At the mid-treatment timepoint, 8/10 patients had undetectable ctHPV16 DNA, which correlated with complete remission of disease. Two patients with residual ctHPV16 DNA at this timepoint had partial regression (n = 1) or clinical progression (n = 1). At the end-of-treatment timepoint, a further six patients had undetectable ctHPV16 DNA, while one patient had ongoing residual ctHPV16 DNA, which correlated with disease progression. Similarly, Lefevre et al. (2021) also monitored plasma HPV levels at different timepoints during treatment [17]. Twelve patients underwent ‘fast elimination’ of plasma HPV, achieving undetectable levels at the mid-treatment timepoint. No patients in this group experienced local or distant treatment failure. A further 20 patients underwent ‘slow elimination’ of plasma HPV, achieving undetectable levels at the end of treatment. Four of twenty patients in this group experienced local treatment failure, and 0/20 patients experienced distant treatment failure. Thirteen patients had persistent detection of plasma HPV at the end of treatment, of which 0/13 experienced local treatment failure, and 4/13 experienced distant treatment failure. All patients with distant treatment failure demonstrated a further increase in plasma HPV at the extended follow-up timepoint.

Bernard-Tessier et al. observed that in their cohort of 36 patients with stage III–IV SCCA treated with 5 months of chemotherapy alone, HPV ctDNA was significantly lower after treatment than at baseline (*p* < 0.001) [14]. Overall, 14/36 patients had residual HPV ctDNA post-treatment (detection rate 38.9%, 95% CI: 24.8–55.1). Baseline ctDNA levels were not associated with tumour response (*p* = 0.79). For patients in this cohort who underwent complete or partial remission, ctDNA level changes pre- and post-treatment were significantly associated with tumour response (*p* < 0.008, *p* < 0.0004, respectively). Ruano et al. observed that among nine patients with paired circulating tumour cell samples pre- and post-treatment, three patients demonstrated a decrease in CTCs/mL, and six patients demonstrated an increase in CTCs/mL [20]. HPV DNA was detected in circulating tumour cells in 14/15 patients pre-treatment (93.33%) and 7/9 patients post-treatment (77.7%). All patients (n = 2) with undetectable HPV DNA post-treatment experienced complete clinical response. Five of seven patients with detectable HPV DNA post-treatment experienced complete clinical response. Azzi et al. observed that 23/27 patients who underwent definitive treatment were ctDNA-negative following treatment [13]. Three of four patients with residual ctDNA positivity experienced disease progression, while 1/23 ctDNA-negative patients post-treatment experienced disease progression. See Table 3 for a summary of the role of ctDNA and treatment response.

### 3.5. ctDNA and Patient Outcomes

Six out of eight included studies observed a correlation between ctDNA and patient survival metrics [13,20]. Three studies identified a correlation with pre-treatment ctDNA status [16,17,18], two with post-treatment ctDNA status [13,15], and one with both pre- and post-treatment ctDNA status [14]. Lefevre et al. (2020) did not identify a correlation between cfDNA changes during treatment and patient prognosis; however, a difference in disease-free survival (DFS) was observed among patients with baseline circulating free DNA levels above the 25th percentile [16]. Similarly, Lefevre et al. (2021) identified a difference in DFS with an HR of 4.07 (CI 0.84–19.64, *p* = 0.08) in addition to a difference in overall survival (OS) with an HR of 2.42 (CI 0.44–13.44, *p* = 0.31) between patients with high versus low median pre-treatment plasma HPV levels [17]. 

Mazurek et al. compared survival metrics between patients who were ctHPV16 DNA-positive and -negative pre-treatment [18]. In this analysis, ctHPV16 DNA status was not a significant parameter affecting overall survival. In multivariate Cox analysis, however, pre-treatment ctHPV16 DNA positivity was a good prognostic factor for DFS without significance (*p* = 0.096). Additional subgroup analysis of patients with a high standardised uptake value (SUVmax) (SUVmax > 13.6) identified pre-treatment ctHPV16 DNA status as an independent prognostic factor within this cohort (*p* = 0.022), with a worse survival outcome for patients who were ctHPV16 DNA-negative prior to treatment (36% survival if node-negative, 0% survival if node-positive).

Azzi et al. included 27 patients in their analysis of ctDNA positivity and survival metrics [13]. They observed that anytime ctDNA positivity post-treatment was associated with significantly shorter DFS (median DFS 11.4 months versus timepoint not reached) (HR 28.0, 95% CI: 2.8–285.0, *p* = 0.005). Cabel et al. analysed paired samples of HPV ctDNA for 18 patients, both pre- and post-treatment, and observed that disease-free survival (DFS) was strongly associated with HPV ctDNA status post-treatment (*p* < 0.0001) [15]. 

Bernard-Tessier et al. did not observe an association between baseline HPV ctDNA positivity and progression-free survival (PFS) [14]. Using receiver operating characteristic (ROC) curve analysis, a baseline HPV ctDNA level of 2940 copies/mL was associated with a sensitivity of 67%, a specificity of 70%, a PPV of 80%, and an NPV of 54% to predict progression following chemotherapy. A baseline HPV ctDNA level below this figure was associated with longer PFS (HR 2.1 (CI 95% 1.0–4.2), *p* = 0.04). Undetectable HPV ctDNA post-treatment was strongly associated with patient outcomes, with a hazard ratio for post-treatment PFS of patients with undetectable HPV ctDNA versus residual HPV ctDNA of 5.5 (95% CI: 2.1–14.3, *p* < 0.001). In multivariate analysis, baseline HPV ctDNA was not associated with survival, while post-treatment HPV ctDNA was a significant predictor of PFS (HR 5.04, 95% CI: 1.9–13.5, *p* = 0.001). The one-year OS rate in patients with undetectable HPV ctDNA was 87%, while the one-year OS rate in patients with residual HPV ctDNA was 50% with an odds ratio of 7 (95%, CI 1.5–28.5, *p* = 0.02). See Table 4 for a summary of the role of ctDNA and patient outcomes.

### 3.6. Risk of Bias

One study was ‘very good’, seven studies were ‘good’, zero studies were “satisfactory”, and zero studies were “unsatisfactory”. Supplementary Material S2 summarises the results of our risk of bias assessment and individual breakdown of included studies.

## 4. Discussion

The results presented in this systematic review highlight the multifaceted role of ctDNA in the context of SCCA. ctDNA holds promise as a new biomarker for both diagnosis and staging of SCCA. Three studies observed greater than 80% sensitivity of ctDNA for HPV-related SCCA, with Bernard-Tessier et al. demonstrating a sensitivity of 91.1% of HPV ctDNA in their cohort of 57 patients with stage III-IV HPV16-related SCCA [14]. Only 1/8 studies, Lefevre et al., documented specificity of ctDNA for SCCA in their cohort (67%) [17]. Importantly, Ellsworth et al. demonstrated that HPV16 cell-free DNA was not detectable in their cohort of patients with either HPV infection or anal HSIL; however, they showed 100% sensitivity for SCCA, highlighting the potential use of HPV16 cell-free DNA as a diagnostic biomarker of HPV-related SCCA [19]. 

Several studies noted a correlation between pre-treatment ctDNA levels and SCCA disease burden, suggesting that ctDNA could play a role as a biomarker for the staging of SCCA. Lefevre et al. (2020) and Lefevre et al. (2021) observed a trend in DNA biomarker levels and advancing tumour stage; however, the trend was not statistically significant in either study [16,17]. Three studies observed a statistical difference between ctDNA levels in patients with lymph node-positive versus lymph node-negative disease [15,17,18], and a further two studies identified a significant difference between patients with metastatic disease versus localised disease [13,14]. Increasing levels of ctDNA could, therefore, prove invaluable as an efficient method of SCCA disease stratification.

There is also evidence for the use of ctDNA to monitor treatment response and treatment failure, as has previously been described in pancreatic adenocarcinoma [21]. Seven of eight studies included in this systematic review examined patient cohorts with paired ctDNA samples pre- and post-treatment, in addition to further timepoints, including mid-treatment and extended post-treatment follow-up. Several studies identified the presence of residual ctDNA post-treatment as a potential indicator of treatment failure. Cabel et al. observed that 3/18 patients were found to have residual ctDNA levels post-treatment, of which all went on to experience metastatic relapse [15]. Conversely, only 1/15 patients with undetectable ctDNA levels post-treatment went on to experience local relapse. Similarly, Azzi et al. observed that 4/27 patients were identified as ctDNA-positive post-treatment, of whom 3/4 experienced disease progression, while only 1/23 patients who were ctDNA-negative experienced disease progression [13]. It has previously been shown that ctDNA may alleviate unnecessary treatment in certain patient cohorts in the post-operative setting [22]. 

Mazurek et al. and Lefevre et al. (2020) both included additional ctDNA measurement timepoints [16,18]. Interestingly, both studies identified groups of patients who achieved undetectable levels of ctDNA at a mid-treatment timepoint, named the ‘fast elimination’ group by Lefevre et al., who noted that 0/12 patients in this cohort experienced local or distant treatment failure [16]. Similarly, Mazurek et al. observed that all eight patients with undetectable ctDNA levels at this timepoint achieved complete disease remission. Mazurek et al. noted that 1/2 patients with residual ctDNA at this timepoint went on to experience disease progression, while a further patient with residual ctDNA at the post-treatment timepoint also went on to experience disease progression [18]. 

Across all seven studies with paired samples, both pre- and post-treatment, a trend was seen towards decreasing ctDNA levels post-treatment; however, the significance of this trend varied, and the implication of this trend in terms of treatment response or treatment failure was not always clear. However, this review has identified that residual ctDNA levels post-treatment may correlate with treatment failure. Furthermore, this systematic review has also identified the presence of a ‘fast elimination’ group who achieve undetectable levels of ctDNA prior to the end of treatment, as this group appear to be less likely to experience treatment failure and may also benefit from a shortened CRT course to prevent unnecessary adverse effects associated with treatment.

Three studies observed a significant difference in DFS/PFS between patients with high versus low levels of pre-treatment ctDNA [14,16,17,23]. Mazurek et al. notably identified that patients who were ctHPV16 DNA-positive prior to treatment had a better survival outcome versus patients who were ctHPV16-negative [18]. Additionally, Bernard-Tessier et al. observed a substantial difference in overall survival between patients with undetectable ctDNA levels post-treatment (87%) and those with residual ctDNA (50%) [14].

Despite ctDNA clearly having potential for implementation in the management of SCCA, we note that there remains a paucity of data regarding the real-world use of ctDNA as a tool to inform clinical decisions. We acknowledge that, to date, most studies like those included in this review were small cohorts. This is reflective of the rarer nature of this neoplasm. In addition, the overall response to CRT for SCCA is good; therefore, large numbers are needed to validate its accuracy of treatment failure. Heterogeneity in terms of study design and methodology precluded the use of meta-analysis in this systematic review. Studies varied significantly in the evaluation of different DNA biomarkers, quantitative versus qualitative ctDNA monitoring, ctDNA monitoring timepoints during treatment, treatment options, and disease stage of included participants. Ultimately, large-scale standardised and collaborative assessments will be needed to better define its future role in the management of SCCA.

## 5. Conclusions

In conclusion, our systematic review underscores the significant promise of circulating tumour DNA (ctDNA) as a diagnostic and prognostic biomarker for patients with SCCA. By synthesising the evidence from the included studies, we have demonstrated the potential utility of both pre- and post-treatment ctDNA in facilitating earlier detection of treatment failure or recurrence in SCCA patients.

## Figures and Tables

**Table 1:** Patient characteristics and treatments.

Author	Year	No of Patients	Median Age (Range)	Disease Stage	Treatment	ctDNA Measurement Timepoints	Assessment of Treatment Response	Median Follow-Up
Ellsworth [19]	2023	40	55.5 (44.8, 61.2)	Anal HSIL	N/A	N/A	N/A	N/A
Mazurek [18]	2023	62	63 (19, 83)	I–III (25 N+, 36 N−)	CRT	baseline, mid-treatment, post-treatment, 1–8 month follow-up, 9–25 month follow-up, 3-year follow-up	RECIST criteria	N/A
Ruano [20]	2022	15	61 (43, 73)	I–III (10 N+, 5 N−)	CRT	baseline (15), post-treatment (9)	N/A	22.2
Lefevre [17]	2021	88	62 (26, 84)	I–IV (21 N+, 1 M+)	CRT	baseline (73), mid-treatment (72), post-treatment (64), 1–3 year follow-up (41)	N/A	29
Lefevre [16]	2020	80	63 (26, 84)	I–IV (21 N+, 1 M+)	CRT	baseline (73), mid-treatment (74), post-treatment (67), 1-year follow-up (29)	N/A	22
Cabel [15]	2018	33	64 (47, 82)	II–III (21 II, 22 III)	CRT	baseline (33), post-treatment (18)	Clinical evaluation, PET-CT, pelvic MRI, CT-TAP	30
Azzi [13]	2018	251	63.5 (27.9, 89.4)	I–IV (30 I, 68 II, 96 III, 49 IV)	CRT (35), immunotherapy (6), radiotherapy (2), chemotherapy (4), chemoimmunotherapy (2), surgery (2)	baseline (243), post-treatment (30)	RECIST criteria	21 (n = 37 subcohort)
Tessier [14]	2018	59	60 (38, 78)	III–IV (6 III, 53 IV)	chemotherapy	baseline (52), chemotherapy completion (36)/discontinuation (8)	RECIST criteria	N/A

N/A: not applicable, CRT: chemoradiation, HSIL: high-grade squamous intraepithelial lesion, N: node, RECIST: Response Evaluation Criteria in Solid Tumours, CT: computed tomography, PET: positron emission tomography, MRI: magnetic resonance imaging.

**Table 2:** The role of ctDNA in SCCA diagnosis and staging.

Study	Year	DNA Biomarker	Detection Method for ctDNA	Sensitivity for SCCA	Specificity for SCCA	Quantitative Pre-Treatment ctDNA Levels and Correlation with Disease Burden
Ellsworth [19]	2023	HPV cell-free DNA	ddPCR	-	100% (95% CI: 91–100%)	-
Mazurek [18]	2023	circulating tumour HPV16 DNA	quantitative PCR	-	-	Correlation between lymph node positivity and higher ctDNA levels (*p* = 0.031)
Ruano [20]	2022	circulating tumour cells, HPV DNA	CISH	-	-	-
Lefevre [17]	2021	plasma HPV	ddPCR	82%	67%	Correlation between lymph node positivity and higher ctDNA levels (*p* = 0.02)
Lefevre [16]	2020	circulating free DNA	spectrometry	-	-	Correlation between gross tumour volume and increasing ctDNA levels (*p* < 0.01)
Cabel [15]	2018	HPV ctDNA	ddPCR	88% (95% CI: 72–95%)	-	Correlation between lymph node positivity and higher ctDNA levels (*p* = 0.03)
Azzi [13]	2018	Personalised ctDNA using tumour SNVs	NGS WES and PCR	-	-	Correlation between metastatic disease and higher ctDNA levels (*p* = 0.004)
Bernard-Tessier [14]	2018	HPV ctDNA	ddPCR	91.1% (95% CI: 81.1–96.2%)	-	Correlation between tumour burden and increasing ctDNA levels (*p* = 0.025) Correlation between metastatic disease and higher ctDNA levels (*p* < 0.001)

SNV: single nucleotide variant, HPV: human papillomavirus, PCR: polymerase chain reaction, NGS: next-generation sequencing, WES: whole exome sequencing, DNA: deoxyribonucleic acid, ddPCR: droplet digital PCR, CISH: chromogenic in situ hybridisation, ctDNA: circulating tumour DNA, SCCA: squamous cell carcinoma of the anal canal.

**Table 3:** ctDNA and treatment response.

Study	Year	DNA Biomarker	No. of Patients with Undetectable ctDNA Mid-Treatment (% with Complete Clinical Response)	No. of Patients with Undetectable ctDNA Post-Treatment (% with Complete Clinical Response)	No. of Patients with Residual ctDNA Post-Treatment (% with Treatment Failure)
Ellsworth [19]	2023	HPV cell-free DNA	-	-	-
Mazurek [18]	2023	circulating tumour HPV16 DNA	8 (100%)	6 (100%)	2 (100%)
Ruano [20]	2022	circulating tumour cells, HPV DNA	-	2 (100%)	7 (28.6%)
Lefevre [17]	2021	plasma HPV	12 (100%)	20 (80%)	13 (30.8%)
Lefevre [16]	2020	circulating free DNA	-	-	-
Cabel [15]	2018	HPV ctDNA	-	15 (93%)	3 (100%)
Azzi [13]	2018	personalised ctDNA using tumour SNVs	-	23 (95.7%)	4 (75%)
Bernard-Tessier [14]	2018	HPV ctDNA	-	22	14

HPV: human papillomavirus, DNA: deoxyribonucleic acid, ctDNA: circulating tumour DNA, SNV: single nucleotide variant.

**Table 4:** ctDNA and patient outcomes.

Study	Year	DNA Biomarker	Correlation between ctDNA and Survival Metrics
Ellsworth [19]	2023	HPV cell-free DNA	-
Mazurek [18]	2023	circulating tumour HPV16 DNA	Pre-treatment ctDNA positivity associated with improved DFS in patients with high SUVmax (*p* = 0.022)
Ruano [20]	2022	circulating tumour cells, HPV DNA	-
Lefevre [17]	2021	plasma HPV	High pre-treatment ctDNA levels associated with shorter DFS (HR 4.07, CI 0.84–19.64, *p* = 0.08) High pre-treatment ctDNA levels associated with shorter OS (HR 2.42, CI 0.44–13.44, *p* = 0.31)
Lefevre [16]	2020	circulating free DNA	Pre-treatment ctDNA levels >25th percentile associated with shorter DFS (*p* = 0.05)
Cabel [15]	2018	HPV ctDNA	ctDNA positivity post-treatment associated with shorter DFS (*p* < 0.0001)
Azzi [13]	2018	personalised ctDNA using tumour SNVs	Anytime ctDNA positivity post-treatment associated with shorter DFS (HR 28.0, 95% CI: 2.8–285.0, *p* = 0.005)
Bernard-Tessier [14]	2018	HPV ctDNA	Pre-treatment ctDNA level < 2940 copies/mL associated with longer PFS (HR 2.1, CI 95% 1.0–4.2, *p* = 0.04) Post-treatment ctDNA positivity associated with shorter PFS (HR 5.5, 95% CI: 2.1–14.3, *p* < 0.001) Post-treatment ctDNA positivity associated with reduced OS (OR 7, 95%, CI 1.5–28.5, *p* = 0.02)

DFS: disease-free survival, SUVmax: maximum standardised uptake value, OS: overall survival, PFS: progression-free survival, HR: hazard ratio, CI: confidence interval, ctDNA: circulating tumour DNA, HPV: human papillomavirus, DNA: deoxyribonucleic acid, OR: odds ratio.

## Supplementary Materials

The following supporting information can be downloaded at: https://www.mdpi.com/article/10.3390/ijms25074005/s1.
