# Supplementary material for: Assessing Circulating Tumour DNA (ctDNA) as a Biomarker for Anal Cancer Management: A Systematic Review
Source: Int J Mol Sci. 2024 Apr 3;25(7):4005. doi: 10.3390/ijms25074005 (PMC11012625; doi:10.3390/ijms25074005)
Supplement: Supplementary file 1 [file ijms-25-04005-s001.zip › ijms-2904314-supplementary.pdf]

Review

Assessing Circulating Tumor DNA (ctDNA) as a Biomarker in Anal Cancer Management: A Systematic Review

Hugo C. Temperley [1,2,3,†], Timothy Fannon [2,†], Niall J. O'Sullivan [1], Maeve O'Neill [2], Benjamin M. Mac Curtain [2], Charles Gilham [4], Jacintha O'Sullivan [5], Grainne O'Kane [6], Brian J. Mehigan [2], Sharon O'Toole [5], John O. Larkin [2], David Gallagher [6,7], Paul McCormick [2] and Michael E. Kelly [2,3,*]

1 Department of Radiology, St. James's Hospital, D08 NHY1 Dublin, Ireland
2 Department of Surgery, St. James's Hospital, D08 NHY1 Dublin, Ireland
3 Trinity St James's Cancer Institute, D08 NHY1 Dublin, Ireland
4 Department of Radiation Oncology, St. James's Hospital, D08 NHY1 Dublin, Ireland
5 Trinity Translational Medicine Institute, Trinity St James's Cancer Institute, Trinity College, St James's Hospital, D08 NHY1 Dublin, Ireland
6 Department of Medical Oncology, St. James's Hospital, D08 NHY1 Dublin, Ireland
7 Department of Genetics, St. James's Hospital, D08 NHY1 Dublin, Ireland
* Correspondence: kellym11@tcd.ie
† These authors contributed equally to the manuscript.

Supplementary material S1: Search strategy

("circulating tumor DNA" OR ctDNA OR "liquid biopsy" OR "plasma DNA" OR "cell-free DNA") AND ("anal cancer" OR "anal carcinoma" OR "anal neoplasm" OR "anal squamous cell carcinoma") AND ("biomarker*" OR "tumor marker*" OR "prognostic marker*" OR "predictive marker*" OR "diagnostic marker*") AND ("management" OR "treatment" OR "therapy" OR "intervention" OR "care")

**Author**	**Selection**				**Comparability**	**Outcome**			**Quality**
	**Representativeness of the exposed cohort**	**Sample size (<25 = no star)**	**Non-respondents**	**Ascertainment of the exposure**	**The subjects in different outcome groups are comparable**	**Assessment of outcome**	**Statistical test**	**Period (<4weeks)**	
**Azzi**	✸	✸	/	✸	✸	/	✸	✸	6
**Tessier**	✸	✸	/	✸	✸	✸	✸	✸	7
**Cabel**	✸	✸	/	/	✸	✸	✸	✸	6
**Lefevre**	✸	✸	✸	/	✸	/	✸	✸	6
**Lefevre**	✸	/	/	/	✸	/	✸	✸	4
**Mazurek**	✸	✸	✸	/	✸	/	✸	✸	6
**Ruano**	✸	✸	/	/	✸	✸	✸	✸	6
**Ellsworth**	✸	/	/	✸	✸	/	✸	✸	5

Supplementary material S2: Risk of Bias assessment - Newcastle-Ottawa scale
